# Investigation of the Anticancer Activity of Coordination-Driven Self-AssembledTwo-Dimensional Ruthenium Metalla-Rectangle

**DOI:** 10.3390/molecules24122284

**Published:** 2019-06-19

**Authors:** Harsh Vardhan, Ayman Nafady, Abdullah M. Al-Enizi, Khalid Khandker, Hussein M. El-Sagher, Gaurav Verma, Mildred Acevedo-Duncan, Tawfiq M. Alotaibi, Shengqian Ma

**Affiliations:** 1 Department of Chemistry, University of South Florida, 4202 East Fowler Avenue, Tampa, FL 33620, USA; hvardhan@mail.usf.edu (H.V.); kmkhalid@mail.usf.edu (K.K.); gauravv@mail.usf.edu (G.V.); macevedo@usf.ed (M.A.-D.); 2 Department of Chemistry, College of Science, King Saud University, Riyadh 11451, Saudi Arabia; amenizi@ksu.edu.sa; 3 Chemistry Department, Faculty of Science, Sohag University, Sohag 82524, Egypt; omran1st@yahoo.com; 4 King Abdullah City for Atomic and Renewable Energy, Riyadh 11451, Saudi Arabia; t.otaibi@energy.gov.sa

**Keywords:** self-assembly, ruthenium complex, amide linker, anticancer activity, cisplatin

## Abstract

Coordination-driven self-assembly is an effective synthetic tool for the construction of spatially and electronically tunable supramolecular coordination complexes (SCCs), which are useful in various applications. Herein, we report the synthesis of a two-dimensional discrete metalla-rectangle [(*η*^6^-*p*-cymene)_4_Ru_4_(C_6_H_2_O_4_)_2_(**2**)_2_](CF_3_SO_3_)_4_ (**3**) by the reaction of a dinuclear half-sandwich ruthenium (II) complex [Ru_2_(*η*^6^-*p*-cymene)_2_(C_6_H_2_O_4_)Cl_2_] (**1**) and bis-pyridyl amide linker (**2**) in the presence of AgO_3_SCF_3_. This cationic ruthenium metalla-rectangle (**3**) has been isolated as its triflate salt and characterized by analytical techniques including elemental analysis, Fourier-transform infrared spectroscopy (FT-IR), proton nuclear magnetic resonance spectroscopy (^1^H-NMR), carbon nuclear magnetic resonance spectroscopy (^13^C-NMR), ^1^H-^1^H correlation spectroscopy (COSY), ^1^H-^1^H nuclear Overhauser effect spectroscopy (NOESY), diffusion ordered spectroscopy (DOSY), and high-resolution electrospray ionization mass spectrometry (HR-ESI-MS). Significantly, the 2D cationic ruthenium metalla-rectangle showed better anticancer activity towards three different cell lines (A549, Caki-1 and Lovo) as compared with the parent ruthenium complex (**1**) and the commercially used drug, cisplatin.

## 1. Introduction

Self-assembly is a vital synthetic tool for the construction of discrete two-dimensional and three-dimensional architectures. The design and study of discrete supramolecular coordination structures is an interesting field of research because of the ease in synthesis of these thermodynamically favored complex structures [1,2,3,4,5,6,7,8,9,10,11,12,13,14,15]. Over the past few decades, the impulse behind the synthesis of self-assembled discrete architecture revolves around practical applications such as catalysis [16,17,18,19,20,21,22], biomedicine [23,24,25,26,27,28,29], and host–guest systems [30,31,32]. Due to the potent physiochemical properties, half-sandwich ruthenium, iridium, and rhodium molecular clips are predominantly employed by various research groups in the synthesis of discrete architectures for a plethora of applications [33,34,35,36,37,38,39,40,41,42,43]. Moreover, the dynamic imine chemistry is also widely utilized for the preparation of platonic, Archimedean, stellated, and faceted solids [44]. In particular, sensing of mono-carboxylate or multi-carboxylate anions is of significant importance owing to their crucial role in chemical, biological, and environmental systems [45,46,47,48]. Oxalate, citrate, and tartrate, for instance, are pertinent in the detection of various diseases and are also essential metabolites in Krebs cycle [49,50,51]. Furthermore, organic linkers with different bent angle having hydrogen bonding synthons (-C=O and -N-H) are largely used to synthesize artificial anion receptors. In this context, Stang and Chi research groups extensively reported a range of discrete 2D and 3D ruthenium structures constructed from the principle of directional bonding used as promising anticancer agents; many of them have been employed in in vivo and in vitro studies [52,53,54,55,56,57,58,59,60].

**Scheme 1:**
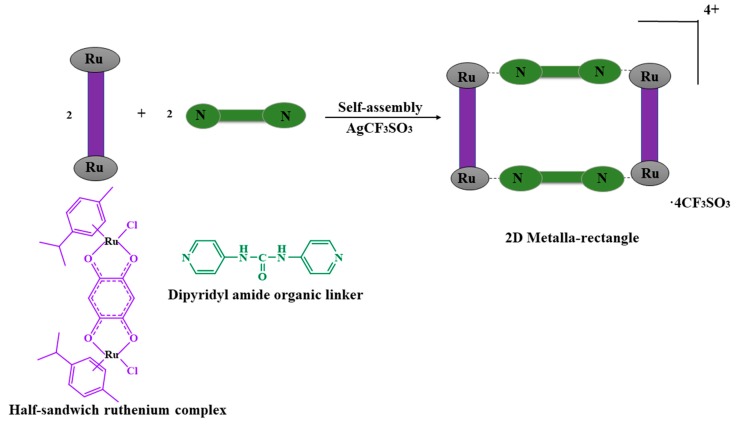
Synthesis of 2D arene ruthenium metalla-rectangle.

Considering the significance and usefulness of the coordination-driven self-assembly approach, herein, a self-assembled arene-Ru derivative has been prepared and screened for its antitumor activity. This may raise the chance of a potential substitute to cisplatin, which has various drawbacks such as the lack of selectivity, high nephrotoxicity, neurotoxicity, and ototoxicity together with the inherent or acquired resistance in various cancer cell lines [61,62,63]. In this paper, we report the synthesis, and characterization of a self-assembled 2D metalla-rectangle [(*η*^6^-*p*-cymene)_4_Ru_4_(C_6_H_2_O_4_)_2_(**2**)_2_](CF_3_SO_3_)_4_ (**3**) prepared by the coordination–driven self-assembly of dinuclear half-sandwich ruthenium (II) complexes [Ru_2_(*η*^6^-*p*-cymene)_2_(C_6_H_2_O_4_)Cl_2_] (**1**) and urea functionalized bis-pyridyl amide linker (**2**) in the presence of AgO_3_SCF_3_ (Scheme 1). The anticancer activity of this compound toward three different cell lines (Lovo, Caki-1, A549) was also tested.

## 2. Results

**Figure 1:**
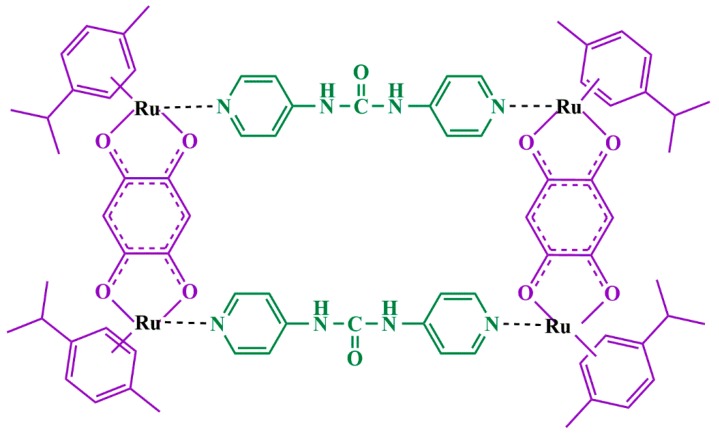
Proposed structure of 2D ruthenium metalla-rectangle (**3**).

Half-sandwich ruthenium complex **1** (Figures S1 and S2) is known for the formation of various discrete architectures based on the symmetry of organic linkers. The reflux reaction of 4-aminopyridine with 1,1′-carbonyldiimidazole in dry tetrahydrofuran (THF) resulted in the formation of dipyridyl organic linker (**2**) as evidenced by Fourier-transform infrared spectroscopy (FT-IR), proton and carbon nuclear magnetic resonance spectroscopy (^1^H-NMR, ^13^C-NMR) (Figures S3 and S4). FT-IR spectrum of **2** showed NH and CO vibrations stretching peaks at 2917 cm^−1^ and 1737 cm^−1^ respectively. ^1^H-NMR spectrum of symmetrical organic linker exhibited one sharp singlet at 9.25 ppm (NH) along with two aromatic protons at 8.35 ppm (CH_α_), 7.42 (CH_β_) ppm and a strong peak at 151.99 ppm in ^13^C-NMR further approves the presence of amide functionalities. Self-assembly of **1** and **2** at a 1:1 ratio using directional bonding approach led to the two-dimensional ruthenium metalla-rectangle (**3**) as its triflate salt (Figure 1). Filtration and precipitation via the addition of diethyl ether yielded an analytically pure solid. Unfortunately, all attempts to grow single crystals for X-ray structural analysis failed. Furthermore, the planarity of urea functionalized bis-pyridyl amide linker and the nature of ruthenium complex **2** further approves the proposed structure of self-assembled metalla-rectangle **3** [64,65,66]. It is worth mentioning that the variation in symmetry elements of organic linkers and ligand to metal complex ratio afford different discrete architectures [67], for instance, self-assembly of pyridine based trigonal planar linker and half-sandwich ruthenium complex in 2:3 ratio led to ruthenium based metalla-prisms [68,69].

The synthesis of discrete arene-ruthenium architectures follows a well-established two-step strategy developed by Süss-Fink [66]. The infrared spectrum of **3** predominantly showed a strong absorption for C-F, C=O, and weak stretching C-H vibrations at 1254 cm^−1^, 1733 cm^−1^, and 3076 cm^−1^, indicating the presence of trifluoromethanesulfonate anions, multiple carbonyl groups, and *p*-cymene moieties, respectively (Figure S5). Similar arene ruthenium based discrete metalla-rectangles, metalla-bowls and metalla-prisms exhibited stretching vibrations in a comparable range for C-F, C=O and C-H functionalities [70,71]. ^1^H-NMR and ^13^C-NMR spectra (Figures S6 and S7) further confirms the successful synthesis of the ruthenium metalla-rectangle. As can be seen in Figure S7, the ^13^C-NMR of **3** exhibited a peak around 184.45 ppm, validating the presence of amide functionality in the discrete structure. Moreover, the ^1^H-NMR of **3** showed proton signal of both ligands and ruthenium complex implying the symmetric coordination driven self-assembly of both precursor units with the NH signal appears as a singlet at 9.11 ppm, while CH_α_ and CH_β_ pyridine protons appear as doublets at 8.00 and 7.53 ppm, respectively. The *p*-cymene groups directly attached to **3** exhibited doublets at 5.92 and 5.70 ppm along with multiplet, singlet, and doublet in the range 2.90–1.30 ppm, which is somewhat similar to free ruthenium complex (**1**). In addition to this, ^1^H-^1^H correlation spectroscopy (COSY), ^1^H-^1^H nuclear overhauser effect spectroscopy (NOESY) were also performed to further confirm the successful synthesis of 2D ruthenium metalla-rectangle (**3**) as shown in Figures S8 and S9. Both ^1^H-^1^H COSY and ^1^H-^1^H NOESY spectrum exhibited the resonance cross peaks to NH, pyridine protons, *p*-cymene moieties in both NMR at specific shift not only corresponds to the successful preparation of only one symmetrical structure but, also discard any possibility of unwanted self-assembled units such as oligomers. The diffusion-ordered NMR (DOSY) spectrum of **3** was also recorded. This technique provides an estimation of the diffusion coefficient of a compound in a solution, which is directly related to the size and shape of the compound [72]. The DOSY experiment confirmed the presence of only one species in solution, with an approximate coefficient and hydrodynamic radius of 4.9 × 10^−10^ m^2^s^−1^ and 0.5 nm respectively (CD_3_NO_2_, 25 °C) (Figure S10). The presence of single diffusion line suggests the formation of one discrete structure exclude the formation of any other side products such as polymers. The peaks are clearly allocated in Figure S10, in general, bigger the assembly, smaller the diffusion coefficient. Similar observations have been observed for analogous arene ruthenium architectures [40,73,74].

**Figure 2:**
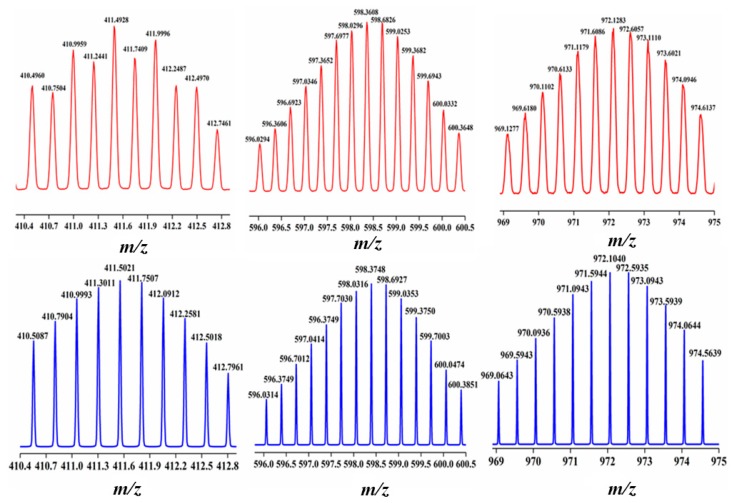
Excerpts of the high-resolution electrospray ionization mass spectrometry (HR-ESI-MS) of the tetracationic (left), tricationic (center), and dicationic (right) species of **3**. Top: experimental, Bottom: theoretical.

The successful formation of tetranuclear **3** was further supported by high resolution electrospray ionization mass spectra (HR-ESI-MS), as shown in Figure S11. Dicationic, tricationic, and tetracationic species corresponding to intact **3** with two, one, and no remaining trifluoromethane sulfonate anions were detected. The HR-ESI-MS spectrum of **3** displayed peak at *m*/*z* 972.1283, 598.3608, and 411.4928, corresponding to [M-2OTf]^2+^, [M-3OTf]^3+^, and [M-4OTf]^4^, respectively. These peaks can be clearly attributed to the 2D metalla-rectangle. The peaks were isotopically resolved and were in good agreement with their theoretical distribution, as shown in Figure 2. More importantly, similar pattern and nature of parent/fragmented ions was also reported by Therrein, Nitschke, Mukherjee, Cook, Stang and Chi research groups for range of discrete ruthenium metalla-assemblies including metalla-rectangles [36,43,70,71,75,76]. We further conducted elemental analysis comparison between ruthenium triflate complex (complex **1** analogous) and complex **3** which in turn, showed good agreement due to presence of triflate counterions as expected (Table S1). In addition, the conductivity study showed a linear variation with increase in concentration of metalla-rectangle **3** and a drastic increase in conductivity of ruthenium triflate complex and metalla-rectangle **3** (168.9 µS) as compared to complex **1** (3.41 µS) was observed validate the presence of triflate counterions in specific proportion (Figure S12) [77]. It is worth to note that these analytical results strongly favor the successful synthesis of proposed 2D discrete metalla-rectangle. Moreover, similar ruthenium-based architectures using amide based organic linker and ruthenium complex were reported with single crystal structure further approved our proposed structure [37,78,79].

**Figure 3:**
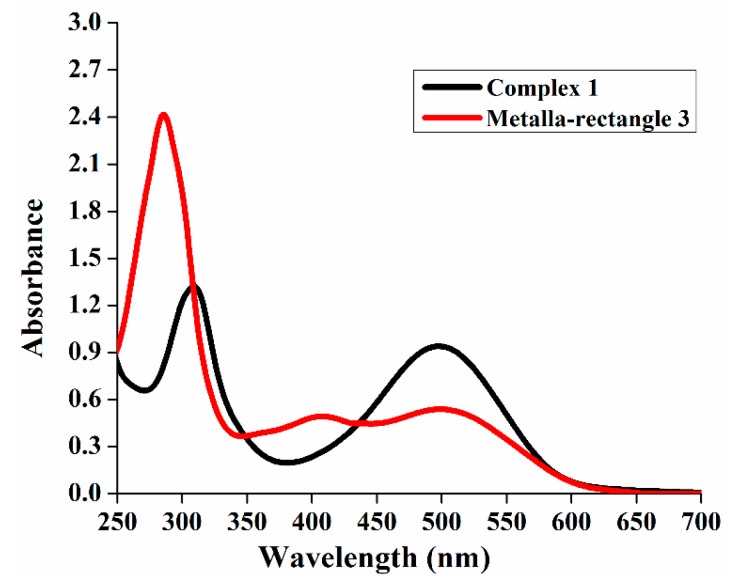
UV-Vis spectra of ruthenium complex (**1**) and 2D metalla-rectangle (**3**) tetracationic in methanol.

Figure 3 showed the electronic absorption spectra of **1** and **3** in methanol (1 × 10^−6^ M). The absorption spectrum of **3** exhibited intense band at 287 and 500 nm. The absorption spectrum of **1** (λ_abs_ = 310 and 501 nm) was similar to that of **3**; however, hypsochromic shift was observed after coordination with the pyridine moieties of **2**. These absorption bands are likely to be an amalgamation of the intramolecular π → π* transitions and metal-to-ligand charge transfer (MLCT) transitions. The complex containing hydrogen bonding synthons are able to interact with a range of anions via the non-covalent interactions. A similar trend has been observed for a range of discrete ruthenium-based architectures [37].

Importantly, the fact that discrete metalla-rectangle **3** contains two bis-amide moieties offers four potential bond donors that can interact, in a specific mode, with multi-carboxylate anions. The behavior of **3** toward biologically important multi-carboxylate anions such as sodium oxalate is shown in Figure S13. UV-Vis absorption spectra of **3** changes significantly upon the addition of increasing concentrations of sodium oxalate owing to the cooperative assistance provided by the hydrogen bonding synthons. As can be seen in Figure S13, metalla-rectangle **3** exhibited two strong absorption bands close to 287 and 502 nm, in which band at 287 nm underwent a bathochromic shift with decreasing intensity, along with the disappearance of a weak intensity shoulder at 404 nm, upon the addition of varying concentrations of sodium oxalate. Moreover, a 1:1 stoichiometry of anion binding to **3** was evident from Job’s plot (Figure S14). A similar observation was reported by Vardhan et. al, who showed the formation of discrete 2D and 3D ruthenium based metalla-rectangle, metalla-bowl, and metalla-prism upon 1:1 binding with multi-carboxylate anions [80,81].

**Figure 4:**
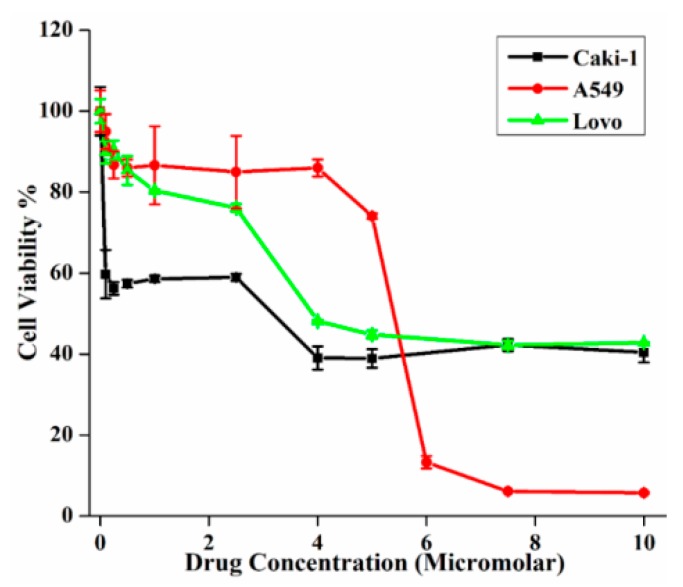
Viability of Caki-1, A549, and Lovo cell lines upon treatment with metalla-bowl (**3**).

Metal-based drugs are widely used in clinical applications. Due to the existing anticancer activity of *O*,*O*-bridged or *N*,*N*-bridged ruthenium supramolecular coordination complexes, the antiproliferative activity of coordination driven self-assembled 2D metalla-rectangle **3** was explored against different cancer cell lines such as A549 (lung cancer), Caki-1 (kidney cancer), and Lovo (colorectal cancer). All the three cancer cell lines were exposed for a particular time to increasing concentrations of **3**, and their activities were determined using WST-1 cell proliferative reagent. The activity of **3** is summarized in Table 1. The IC_50_ values range from 3 to 4 µM for Lovo, Caki-1, A549 cell lines. Fascinatingly, **3** was observed to inhibit the proliferation of all the three cell lines, even at a very low concentration (Figure 4). This finding clearly established that metalla-rectangle **3** was profoundly more effective than cisplatin and ruthenium complex (**1**) in inhibiting the growth of different cancer cell lines and could be a potent candidate as a chemotherapeutic drug against cisplatin-resistant cancer cell lines [27,59,66,82,83]. Notably, the increase in drug concentration led to complete cell kills (Caki-1, Lovo).

Cell apoptosis is an important phenomenon responsible for destroying undesirable cells during the development and homeostasis of cellular organisms [84]. We minutely studied three metastatic cancer cell proliferations and compared them with normal Human Embryonic Kidney cells (HEK 293) as shown in Figure S15. It was observed that cell proliferation for A549, LoVo, and Caki-1 cells reduced to 58%, 66%, and 63%, respectively, at 5.0 µM **3**. However, at the same concentration of **3**, there was practically no reduction for HEK 293, indicating that this potential anticancer drug has almost no toxicity for the normal cells at this particular concentration. Experimental details are described in Materials and methods section. The cancer cells growing rapidly unwind DNA for replication and thus, leave the DNA strands available for drug binding. The drug-DNA complex inhibits the DNA replication and DNA repair, leading to cancer cell destruction and apoptosis. Normal cells have slower growth rate and are less affected by the drug. Therefore, the drug acts only on the rapidly proliferating cells, i.e., cancer cells and specifically inhibits the growth of cancer cells, leaving the normal cells unaffected at a certain concentration. The study of DNA ladder assay is very useful because of its easy availability for the quick screening of apoptotic changes in the cell population. The presence of similar self-assembled structures such as **3** shown DNA ladder assay studies further approve the existence of drug-DNA complex to inhibit DNA replication and repair [42,85,86]. Furthermore, it is expected that incubation with 5.0 µM can severely elevate the apoptotic population of cancer cells.

## 3. Materials and Methods

All chemicals, solvents, deuterated solvents used in this work were purchased from commercial sources and used without purification. Starting materials, arene-ruthenium chloride (**1**) and bent organic linker (**2**), were prepared according to the methods reported in literature [39,55]. 4-amino pyridine (98%; Oakwood Chemicals, West Columbia, SC, USA), 1,1-carbonyldiimidazole (97%; Sigma Aldrich, St. Louis, MI, USA), sodium oxalate (99.9%), dichloro(*p*-cymene)ruthenium(II)dimer (98%; Alfa Aesar, Heysham, Lancashire, UK), 2,5-dihydroxy-1,4-benzoquinone (98%; Sigma Aldrich). ^1^H-NMR, ^13^C-NMR, ^1^H-^1^H COSY, and ^1^H-^1^H NOESY spectra were recorded using an Innova 400 MHz NMR spectrometer (Varian Inc., Palo Alto, CA, USA). ^1^H-NMR chemical shifts are reported relative to residual solvent signals. HR mass spectra were recorded in the positive mode on a Waters Synapt G2 tandem mass spectrometer. Elemental analyses were performed using an Elemental GmbH Vario EL cube (Elementar, Langenselbold, Germany). Absorption spectra were recorded using a JASCO V-670 spectrometer (Jasco Corporation, Hachioji, Tokyo, Japan). Conductivity measurement was performed using Oakton Con 6 Acorn series conductivity meter (Eutech Instruments, Melrose, MA, USA).

Human cancer cell lines, Lovo, Caki-1, and A549 were purchased from ATCC: The Global Bioresource Center (Manassa, VA, USA). Cell proliferation reagent, WST-1, was used. Cells (3500 cells) were plated in triplicates on a 96-well microplate in McCoy’s 5A (Logan, UT, USA) and F12K medium (Manassa, VA, USA). The cells were adhered after 24 h and were treated with different doses. The treatment was continued for 3 consecutive days, and after treatment on the 4^th^ day, 20 µL WST-1 reagent was added to the plates with 180 µL of media and mixed well. Three trials were performed for each cell lines, and IC_50_ values were calculated. The prepared Ru based metalla-bowl (**3**) was tested for its anti-proliferative effect on three different metastatic cancer cell lines, namely, colorectal cancer (LoVo), kidney cancer (Caki-1) and lung cancer (A549). Effect on normal HEK 293 cells was also tested. Using the WST-1 assay, the effect of different drug concentrations on the cell proliferation was found for each cell line. In the 96-well microplate, 3500 cells were plated in each well, and 200 µL cell culture media was added. After 24 h, when the cells were adhered to the bottom of the wells, the media was replaced with fresh media. The cells were then treated with different doses (0.10, 0.25, 0.50, 1.0, 2.5, 5.0, 7.50, and 10.0 µM) of the drug. The control for each cell line was treated with equal volume of DMSO. After 24 h of treatment, the media was again removed and replaced with 180 µL of fresh media. Additionally, 20 µL WST-1 reagents were added to the wells and mixed. The microplates were then incubated in an incubator (37 °C, 5% CO_2_) and read at time t = 0 and t = 2 h. Three trials were performed for each cell line. Drug solutions were prepared using cell culture grade water from a stock 10 mM solution of the Ru-complex in DMSO.

### 3.1. Preparation of Ruthenium Complex (**1**)

A mixture of [Ru_2_(*η*^6^-*p*-cymene)_2_(µ-Cl)Cl]_2_ (184 mg, 0.3 mmol) and 2,5-dihydroxy-1,4-benzoquinone (42 mg, 0.3 mmol) was suspended in 30 mL MeOH and stirred for 2 h at room temperature. The blood red precipitate was filtered, washed with diethyl ether, and dried in vacuo. (Yield: 162 mg, 81%). FTIR (cm^−1^): 1516 (s), 1372 (s), 1257 (s). ^1^H-NMR (CDCl_3_): δ (ppm) 5.78 (s, 2H), 5.60 (d, 4H), 5.37 (d, 4H), 2.88 (sept, 2H), 2.27 (s, 6H), 1.30 (d, 12H). Anal. Calcd for C_26_H_30_Cl_2_O_4_Ru_2_: C, 45.90; H, 4,45. Found: C, 45.67; H, 4.55.

4-Aminopyridine (0.941 g, 10 mmol) was dissolved in 25 mL dry THF. To this solution, 1,1ʹ-carbonyldiimidazole (0.97 g, 6 mmol) was added. The reaction mixture was refluxed for 12 h under inert atmosphere, and the mixture was cooled afterwards. The resulting residue was washed with water several times and dried in vacuo to give a white precipitate. FTIR (cm^−1^): 2917 (N-H), 1737 (C=O). ^1^H-NMR (DMSO-*d*_6_): δ (ppm) 9.25 (s, 2H), 8.35 (d, 4H), 7.42 (d, 4H). ^13^C-NMR (DMSO-*d*_6_): δ (ppm) 151.99, 150.63, 146.40, 112.96.

### 3.2. Synthesis of Metalla-Rectangle (**3**)

A mixture of [Ru_2_(*ƞ*^6^-*p*-cymene)_2_(C_6_H_2_O_4_)Cl_2_] (**1**) (1 mmol) and 2 equivalents of AgCF_3_SO_3_ (2 mmol) in 25 mL methanol was stirred in the dark for 3 h at room temperature. The white precipitate was filtered, and the corresponding red filtrate was added to 1,3-di(pyridin-4-yl)urea (**2**) (1 mmol) and stirred for 15 h; the solvent was removed under reduced pressure. The crude product thus obtained was redissolved in nitromethane and subjected to vapor diffusion of diethyl ether, resulting in a red colored product after several days. Yield (89%). Anal. Calcd for C_78_H_80_O_22_N_8_S_4_F_12_Ru_4_: C, 41.79; H, 3.60; N, 5.00. Found: C, 40.69; H, 3.19; N, 5.12. FTIR (cm^−1^): 3076 (CH_aryl_), 1733 (C=O), 1254 (C-F). ^1^H-NMR (CD_3_NO_2_): δ (ppm) 9.11 (s, 4H; NH), 8.00 (d, 8H, *J* = 6.8 Hz, CH_α_; H_b_), 7.53 (d, 8H, *J* = 6.7 Hz, CH_β_; H_c_), 5.92 (d, 8H, *J* = 6.0 Hz; H_cym_), 5.77-5.69 (m, 12H; H_cym_/H_benz_), 2.89 (sept, 4H; -CH(CH_3_)_2_), 2.18 (s, 12H; -CH_3_), 1.32 (d, 24H, *J* = 6.9 Hz; -CH(CH_3_)_2_). ^13^C-NMR (CD_3_NO_2_): δ (ppm) 184.45, 153.00, 150.36, 148.82, 121.60, 119.69, 114.04, 103.39, 101.88, 98.13, 83.77, 81.04, 30.75, 20.89, 17.10. HR-ESI-MS: *m/z* = 411.4928 [M-4OTf]^4+^, 598.3608 [M-3OTf]^3+^, 971.1283 [M-2OTf]^2+^.

### 3.3. Stability of Metalla-Bowl in DMSO

For the stability analysis, 2D metalla-rectangle was dissolved in DMSO, and the sample was subjected to ^1^H NMR spectroscopy immediately after dissolution and after 3 days. No change in proton NMR was observed, hence confirming the stability of the 2D ruthenium metalla-rectangle.

### 3.4. UV-Vis Binding Studies

A stock solution of metalla-rectangle (**3**) in methanol (1 × 10^−5^ M) and sodium oxalate (1 × 10^−3^ M) in water were prepared. The change in absorbance was recorded at room temperature after the addition of increasing concentrations of sodium oxalate to a constant volume of **3**. The stoichiometry of the adduct with **3** (A-Å) was obtained from these spectra (Job’s plot).

## 4. Conclusions

In conclusion, we have reported the synthesis of coordination driven self-assembled 2D ruthenium metalla-rectangle (**3**) by the reaction of half sandwich ruthenium complex (**1**) and bent organic linker (**2**). Wide range of structural and analytical tools such as FTIR and UV-Vis spectroscopy, elemental analysis, ^1^H NMR, ^13^C NMR, ^1^H-^1^H COSY, ^1^H-^1^H NOESY, and DOSY spectroscopy, HR-ESI-MS spectrometry all confirmed the successful formation of **3**. This new metalla-rectangle showed powerful anticancer activity against three different cell lines (Lovo, Caki-1, A549), as compared with the parent ruthenium precursor and the commercial available cisplatin drug. Importantly, similar self-assembled discrete ruthenium architecture exhibit cell apoptosis therefore future studies are conducting in our laboratory with normal cells and DNA ladder assay provide insights into the cell apoptosis.

## Figures and Tables

**Table 1:** Cytotoxicity comparison of the metalla-rectangle **3** with cisplatin and ruthenium complex **1** in different cancer lines.

Cell Lines	IC_50_, µM^A^	Cisplatin	Ruthenium Complex (1)
**LOVO**	3.2 ± 1.13	7.1 ± 0.67	>100
**CAKI-1**	3.3 ± 1.21	>100	
**A549**	4.0 ± 1.28	>100	>100

^a^ Drug concentration necessary for 50% inhibition of cell viability.

## Supplementary Materials

The following are available online at https://www.mdpi.com/1420-3049/24/12/2284/s1, Figures S1–S15: showing IR, NMR, MS characterizations, Job’s plot and the effect of **3** on difference metastatic and normal cancer lines
