# Supplementary material for: Investigation of the Anticancer Activity of Coordination-Driven Self-AssembledTwo-Dimensional Ruthenium Metalla-Rectangle
Source: Molecules. 2019 Jun 19;24(12):2284. doi: 10.3390/molecules24122284 (PMC6630691; doi:10.3390/molecules24122284)
Supplement: Supplementary file 1 [file molecules-24-02284-s001.pdf]

Electronic Supporting Information

Investigation of the Anticancer Activity of Coordination-Driven Self-Assembled Two-Dimensional Ruthenium Metalla-Rectangle

Harsh Vardhan [1], Ayman Nafady [2,3,*], Abdullah M. Al-Enizi [2], Khalid Khandker [1], Hussein M. El-Sagher [3], Gaurav Verma [1], Mildred Acevedo-Duncan [1], Tawfiq M. Alotaibi [4] and Shengqian Ma [1,*]

[1] Department of Chemistry, University of South Florida, 4202 East Fowler Avenue, Tampa 33620, FL, USA; hvardhan@mail.usf.edu (H.V.); kmkhalid@mail.usf.edu (K.K.); gauravv@mail.usf.edu (G.V.); macevedo@usf.ed (M.A.-D.)

[2] Department of Chemistry, College of Science, King Saud University, Riyadh 11451, Saudi Arabia; anafady@ksu.edu.sa (A.N.); amenizi@ksu.edu.sa (A.M.)

[3] Chemistry Department, Faculty of Science, Sohag University, Sohag 82524, Egypt; omran1st@yahoo.com (H.M.E.-S.)

[4] King Abdullah City for Atomic and Renewable Energy, Riyadh 11451, Saudi Arabia; t.otaibi@energy.gov.sa (T.M.A.)

* Correspondence: anafady@ksu.edu.sa (A.N.); sqma@usf.edu (S.M.); Tel.: +966569407110 (A.N.); +1813-974-5217 (S.M.).

Table of Contents

Figure S1. FT-IR spectrum of half-sandwich ruthenium complex **1**.

Figure S2. ^{1}H-NMR spectrum of ruthenium complex **1** in $CDCl_3$.

Figure S3. FT-IR Spectrum of organic ligand **2**.

Figure S4. ^{1}H-NMR (top) and ^{13}C-NMR (bottom) spectrum of ligand **2** in DMSO-d_6.

Figure S5. FT-IR Spectrum of 2D ruthenium metalla-rectangle **3**.

Figure S6. ^{1}H-NMR spectrum of 2D Metalla-rectangle **3** in CD_3NO_2.

Figure S7. ^{13}C-NMR spectrum of 2D Metalla-rectangle **3** in CD_3NO_2.

Figure S8. ^{1}H-^{1}H NOESY NMR spectrum of 2D Metalla-rectangle **3** in CD_3NO_2.

Figure S9. ^{1}H-^{1}H COSY NMR spectrum of 2D Metalla-rectangle **3** in CD_3NO_2.

Figure S10. DOSY NMR Spectrum of 2D Metalla-rectangle **3** in CD_3NO_2.

Figure S11. HR-ESI-MS Spectra of 2D metalla-rectangle **3** in methanol.

Figure S12. Variation in conductivity of metalla-rectangle **3** with concentration.

Table S1. Elemental analysis comparison of ruthenium triflate complex and metalla-rectangle **3**.

Figure S13. Change in absorbance of metalla-rectangle (**3**) upon addition of varying concentration of sodium oxalate.

Figure S14. Job's plot of oxalate anion titrations with metalla-rectangle **3** showing 1:1 fitting curve.

Figure S15: Effect of metalla-rectangle (**3**) on difference metastatic cancer lines and normal cell lines.

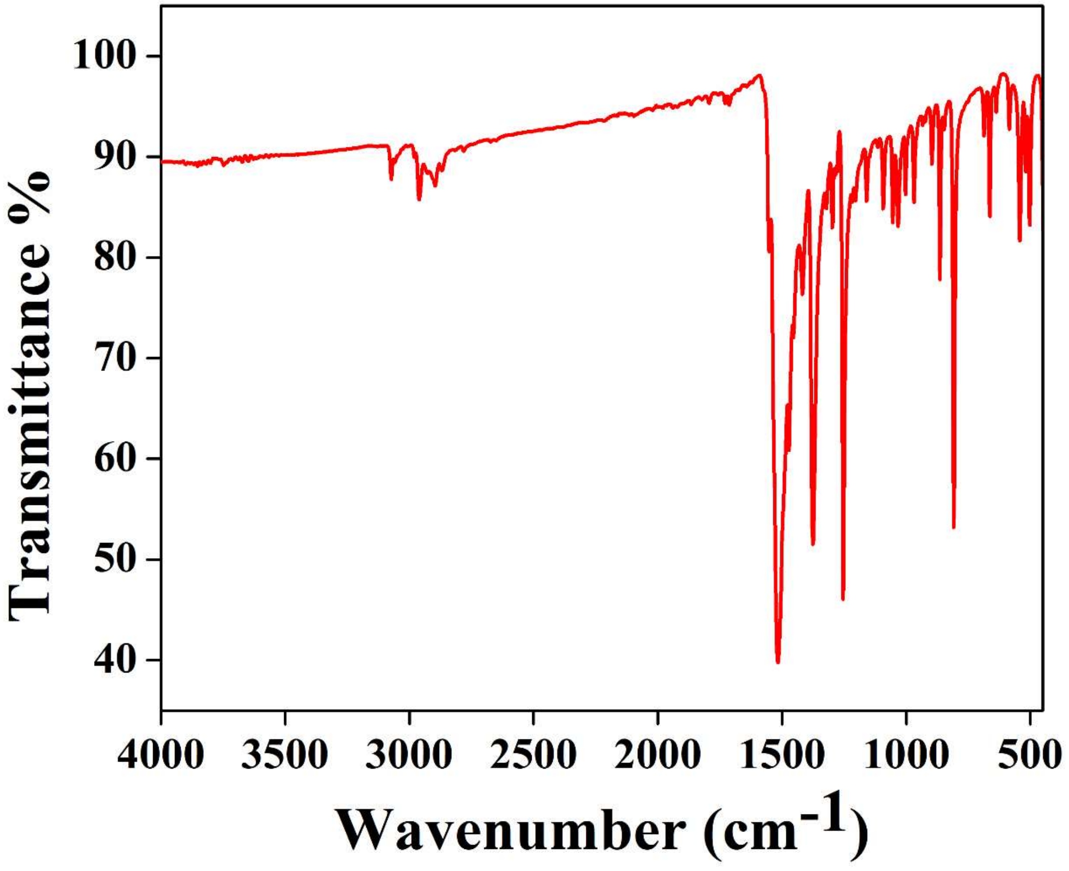

Figure S1. FT-IR spectrum of half-sandwich ruthenium complex **1**.

Peak (cm^{-1})	Assignment for half-sandwich ruthenium complex **1**
3061	C-H mode of vibrations
1516	Aromatic C-O stretch

1372	Aromatic C-C stretch
1257	-C-H breathing
1061	=C-H bend
811	Ru-O stretch

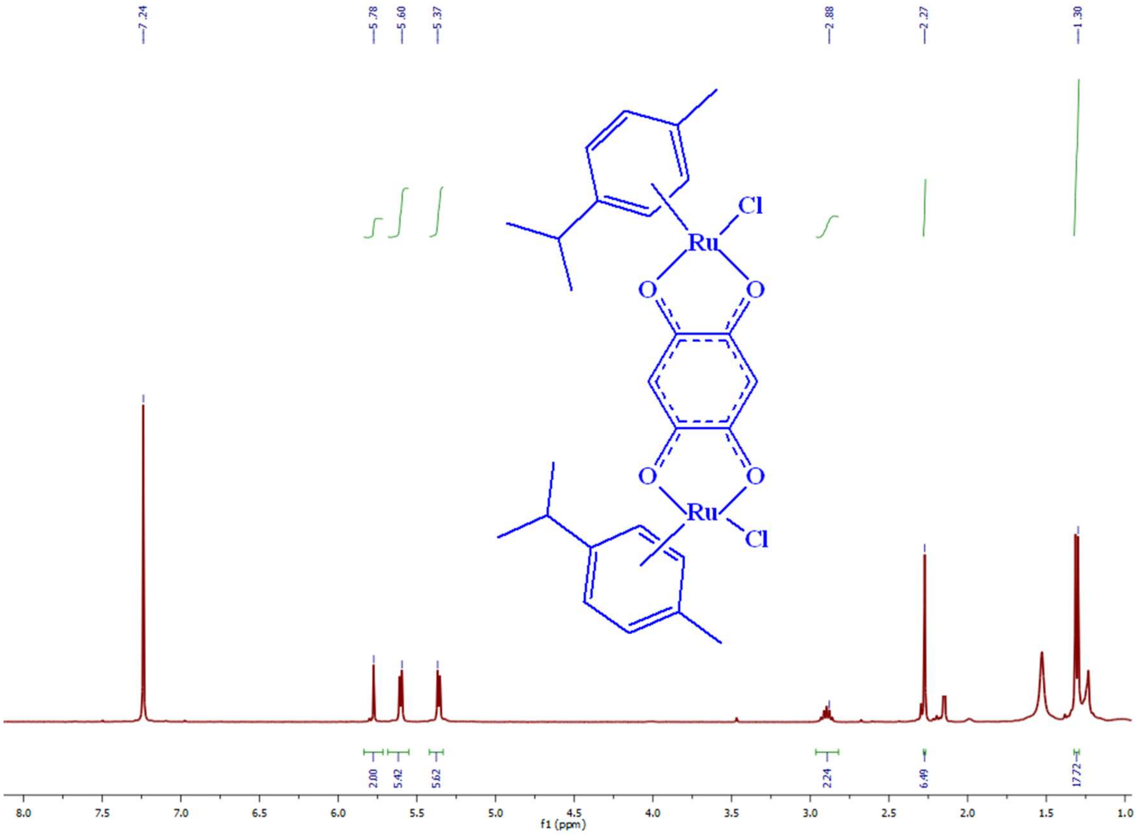

Figure S2. ^{1}H-NMR spectrum of ruthenium complex **1** in $CDCl_3$.

^{1}H NMR (400 MHz, $CDCl_3$): δ (ppm) = 5.78 (s, 2H, Hq), 5.60 (d, 4H, $^{3}J_{H\text{-}H}$ = 6.08 Hz, Har), 5.37 (d, 4H, Har), 2.88 (sept, 2H, $J_{H\text{-}H}$ = 6.68 Hz, CH), 2.27 (s, 6H, CH_3), 1.30 (d, 12H, CH_3);

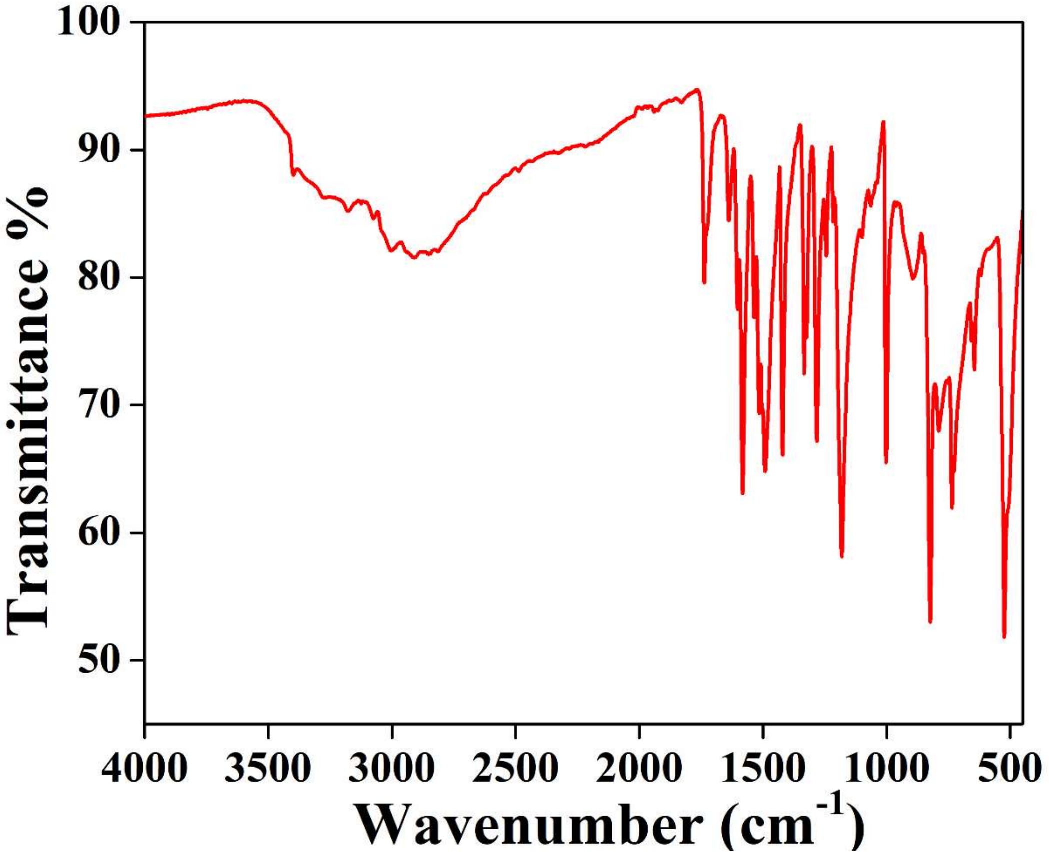

Figure S3. FT-IR Spectrum of organic ligand **2**.

Peak (cm^{-1})	Assignment for bent organic linker **2**
2917	-NH stretch
1737	-C=O stretch
1581	Aromatic C-C stretch
1493	Aromatic C-C stretch
1421	Aromatic C-N stretch
1332	-C-N stretch
1176	-C-H Breathing
1001	=C-H Bend

824	Aromatic C-H out-of-plane bending from phenyl group

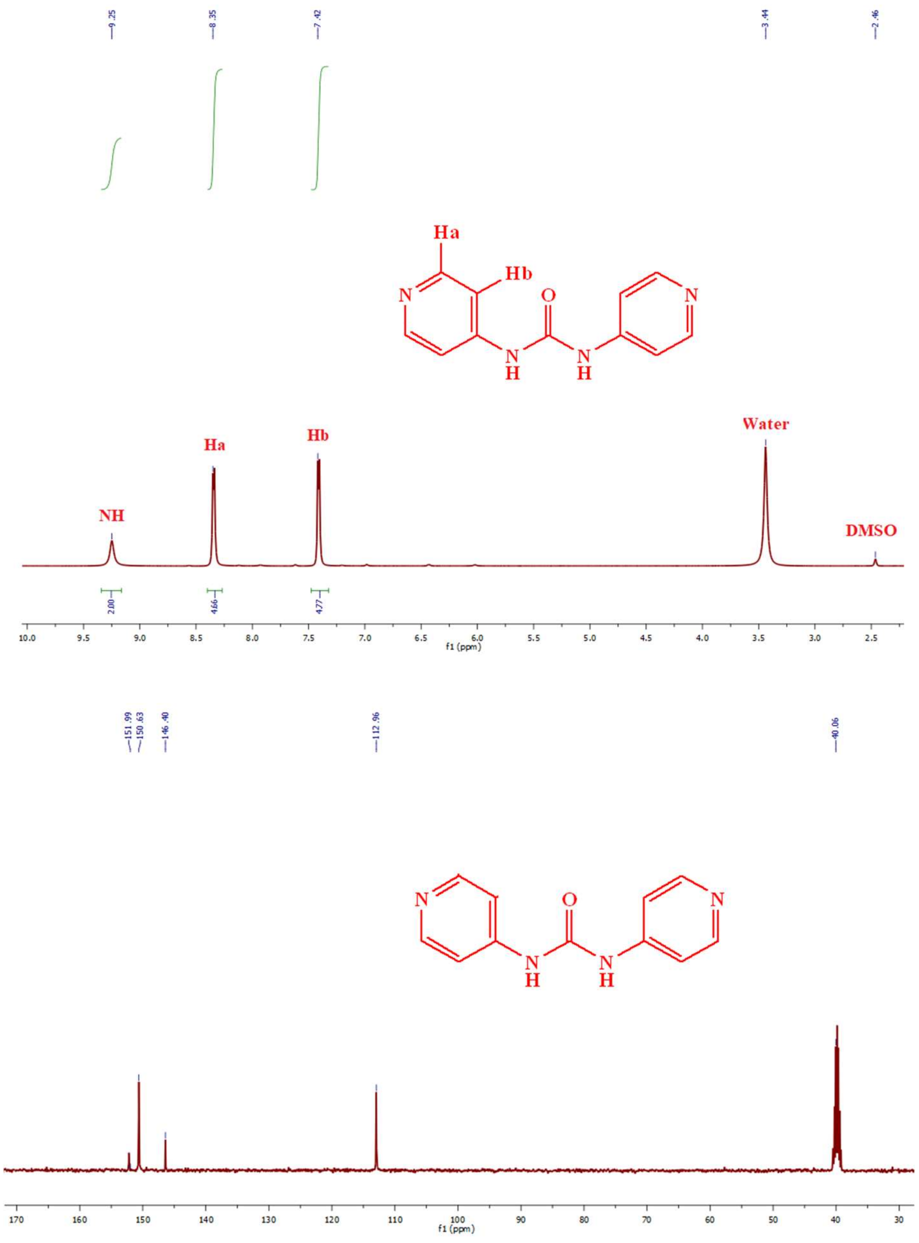

Figure S4. ^{1}H-NMR (top) and ^{13}C-NMR (bottom) spectrum of ligand **2** in DMSO-*d*6.

^{1}H-NMR (400 MHz, DMSO-d_6): δ (ppm) = 9.25 (s, 2H), 8.35 (d, 4H, *J*=7.2 Hz), 7.42 (d, 4H, *J*=7.2 Hz).

^{13}C-NMR (400 MHz, DMSO-d_6): δ (ppm) = 151.99, 150.63, 146.40, 112.96.

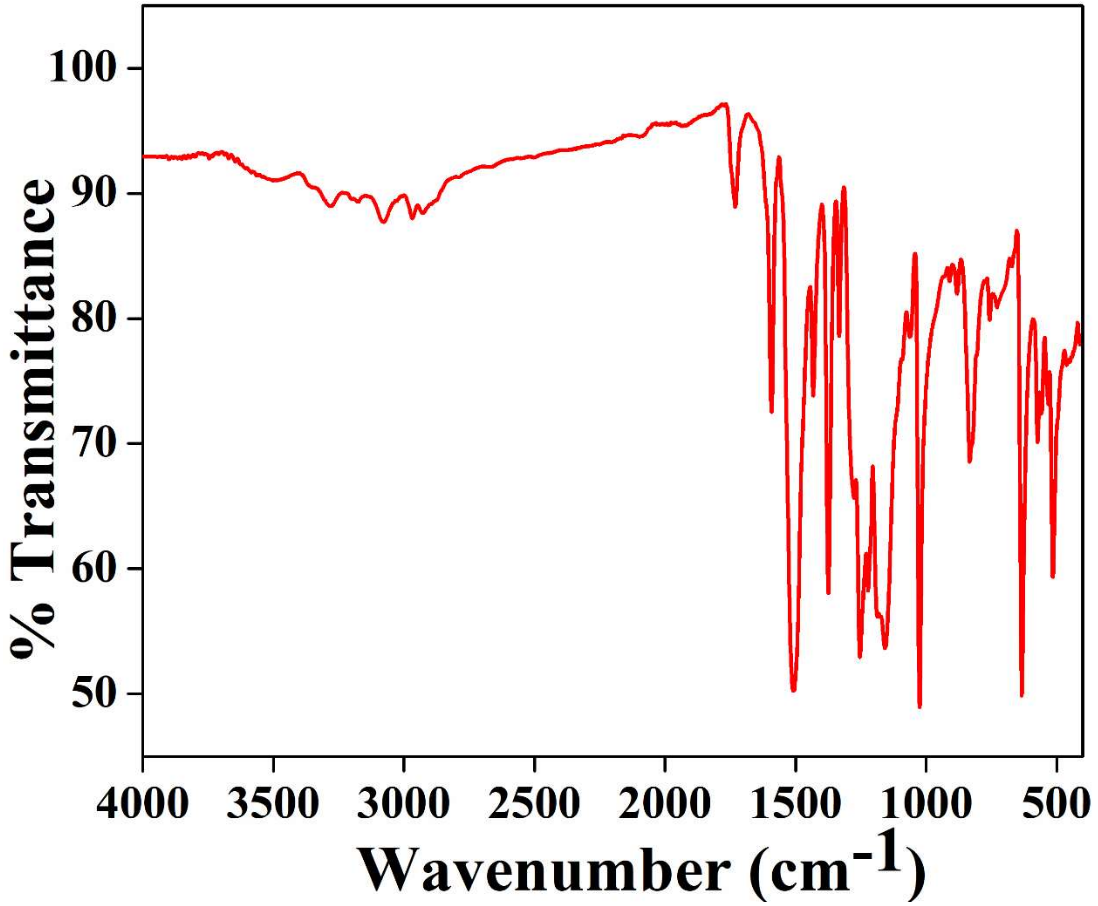

Figure S5. FT-IR Spectrum of 2D ruthenium metalla-rectangle **3**.

Peak (cm^{-1})	Assignment for bent organic linker **2**
3076	(w, CH_{aryl})
1733	-C=O stretch
1593	Aromatic C-C stretch
1507	Aromatic C-C stretch
1376	Aromatic C-N stretch
1254	-C-F stretch

1025	-C-H Breathing
1001	=C-H Bend
827	Aromatic C-H out-of-plane bending from phenyl group

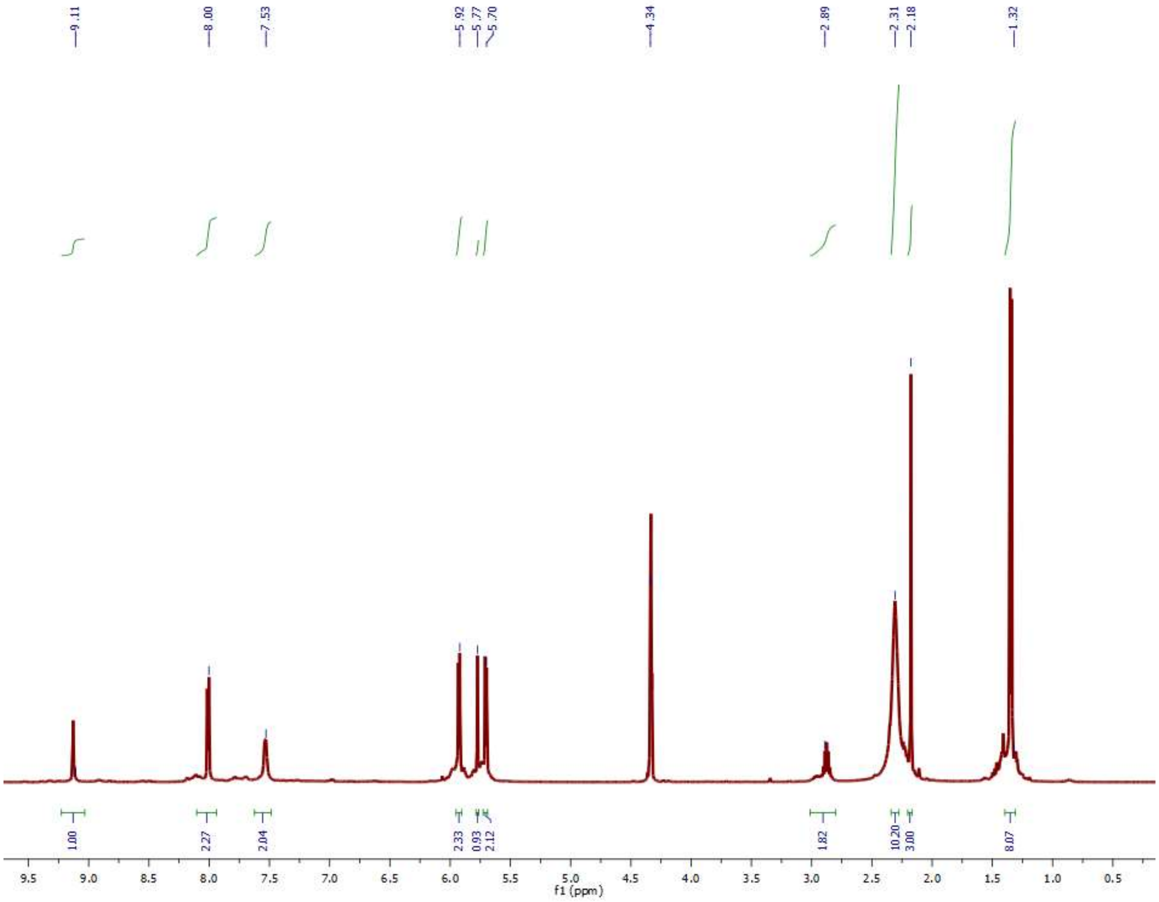

Figure S6. ^{1}H-NMR spectrum of 2D Metalla-rectangle **3** in CD_3NO_2.

^{1}H NMR (CD_3NO_2): δ (ppm) = 9.11 (s, 4H; NH), 8.00 (d, 8H, *J* = 6.8 Hz, CH_α; H_b), 7.53 (d, 8H, *J* = 6.7 Hz, CH_β; H_c), 5.92 (d, 8H, *J* = 6.0 Hz; H_{cym}), 5.77-5.69 (m, 12H; H_{cym}/H_{benz}), 2.89 (sept, 4H; $-CH(CH_3)_2$), 2.18 (s, 12H; $-CH_3$), 1.32 (d, 24H, *J* = 6.9 Hz; $-CH(CH_3)_2$).

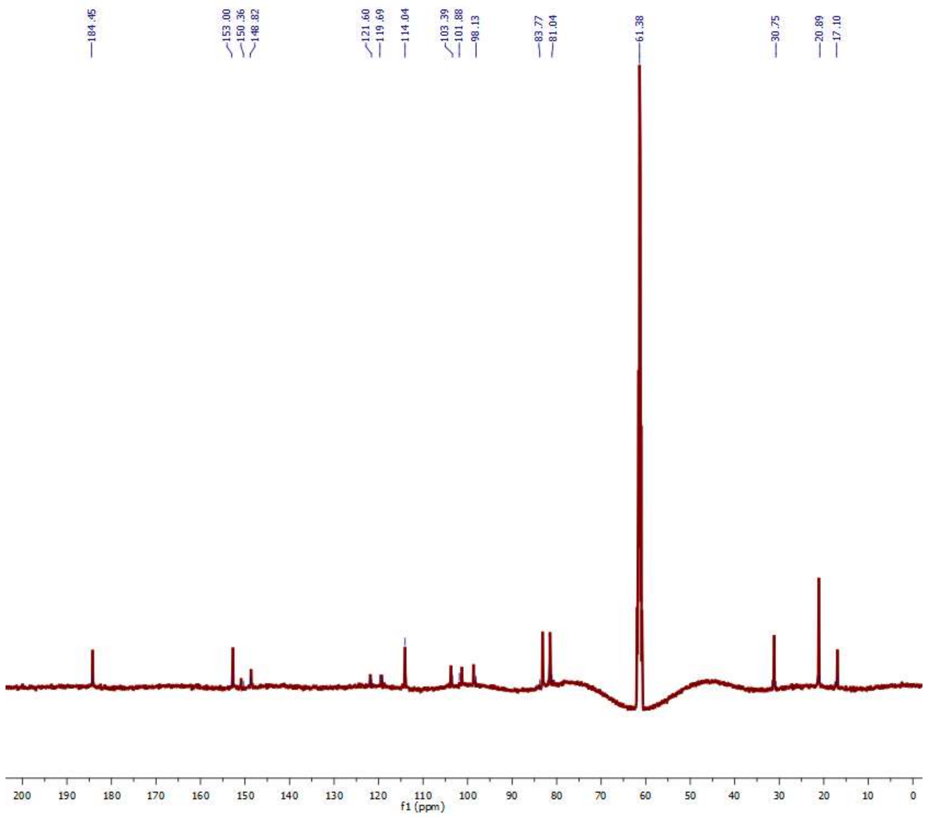

Figure S7. ^{13}C-NMR spectrum of 2D Metalla-rectangle **3** in CD_3NO_2.

^{13}C NMR (CD_3NO_2): δ (ppm) = 184.45, 153.00, 150.36, 148.82, 121.60, 119.69, 114.04, 103.39, 101.88, 98.13, 83.77, 81.04, 30.75, 20.89, 17.10.

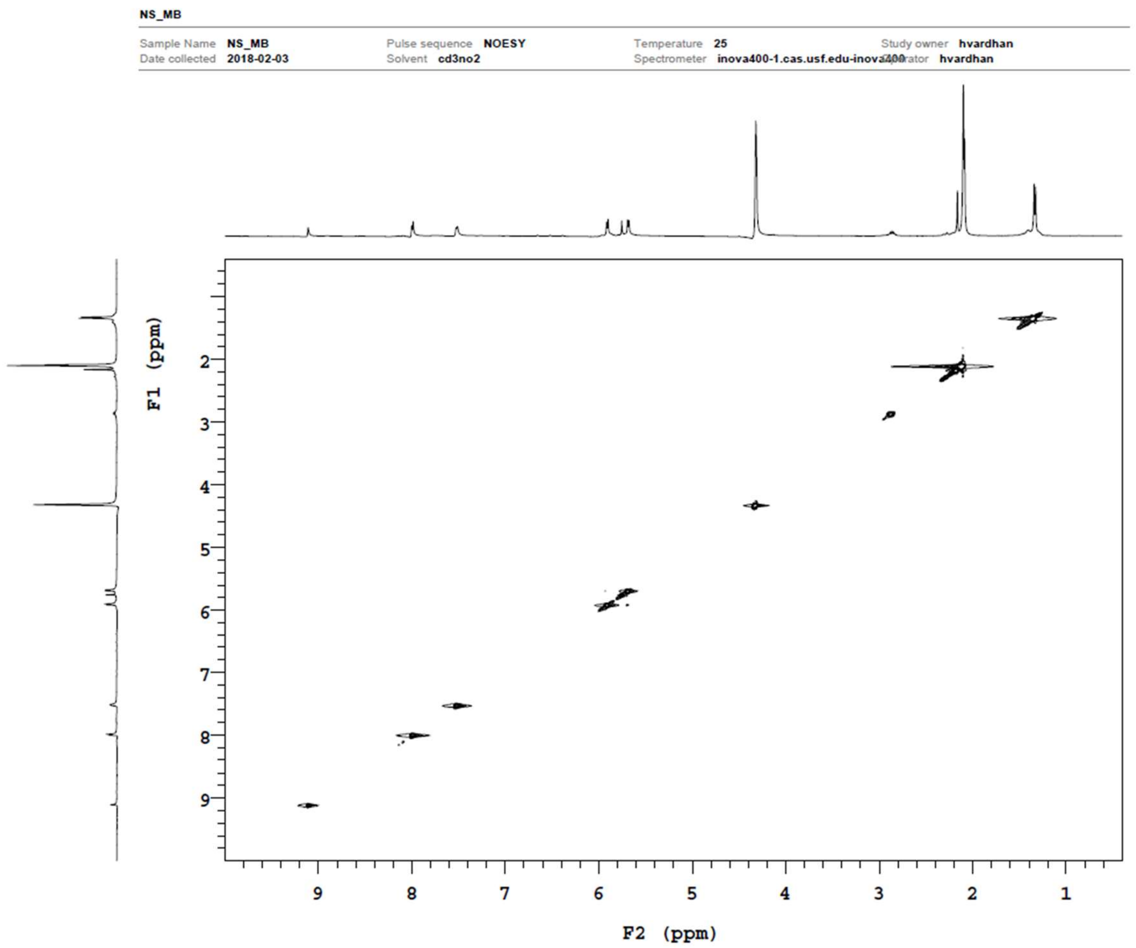

Figure S8. ^1H-^1H NOESY NMR spectrum of 2D Metalla-rectangle **3** in CD_3NO_2.

As mentioned in Figure S6 (^1H-NMR), the NH singlet at 9.11 ppm, pyridine alpha and beta protons at 8.00 ppm and 7.53 ppm respectively along with cymene protons at 5.92 ppm and 5.77-5.69 ppm. The structural correlation as highlighted via the cross peaks imply only one self-assembled symmetrical structures and discard any other structural possibilities.

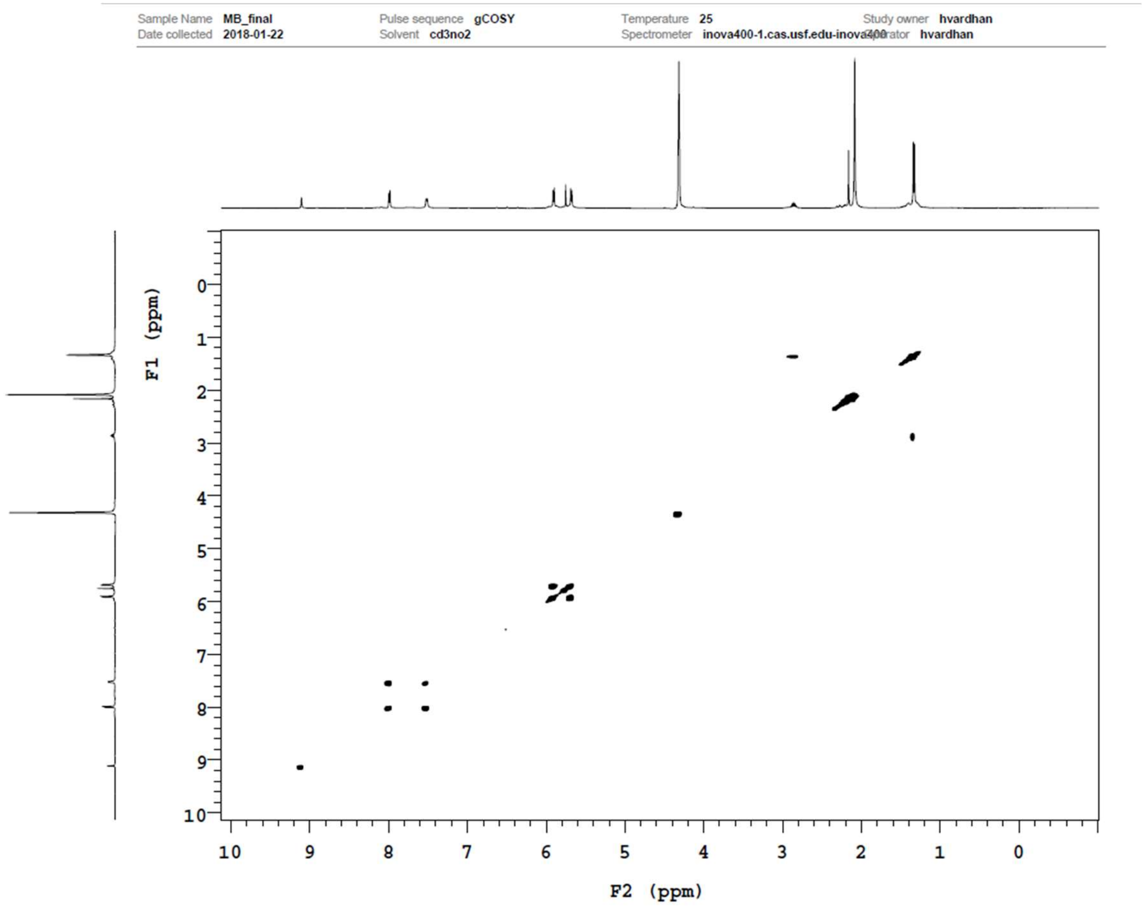

Figure S9. ^{1}H-^{1}H COSY NMR spectrum of 2D Metalla-rectangle **3** in CD_3NO_2.

The ^{1}H-^{1}H COSY NMR spectrum of coordination driven self-assembled ruthenium metalla-bowl **3** shows the cross peaks with chemical shift of 9.11, 8.00, 7.53, 5.92, 5.77, 2.89, 2.18, 1.32 ppm affirm the proposed structure.

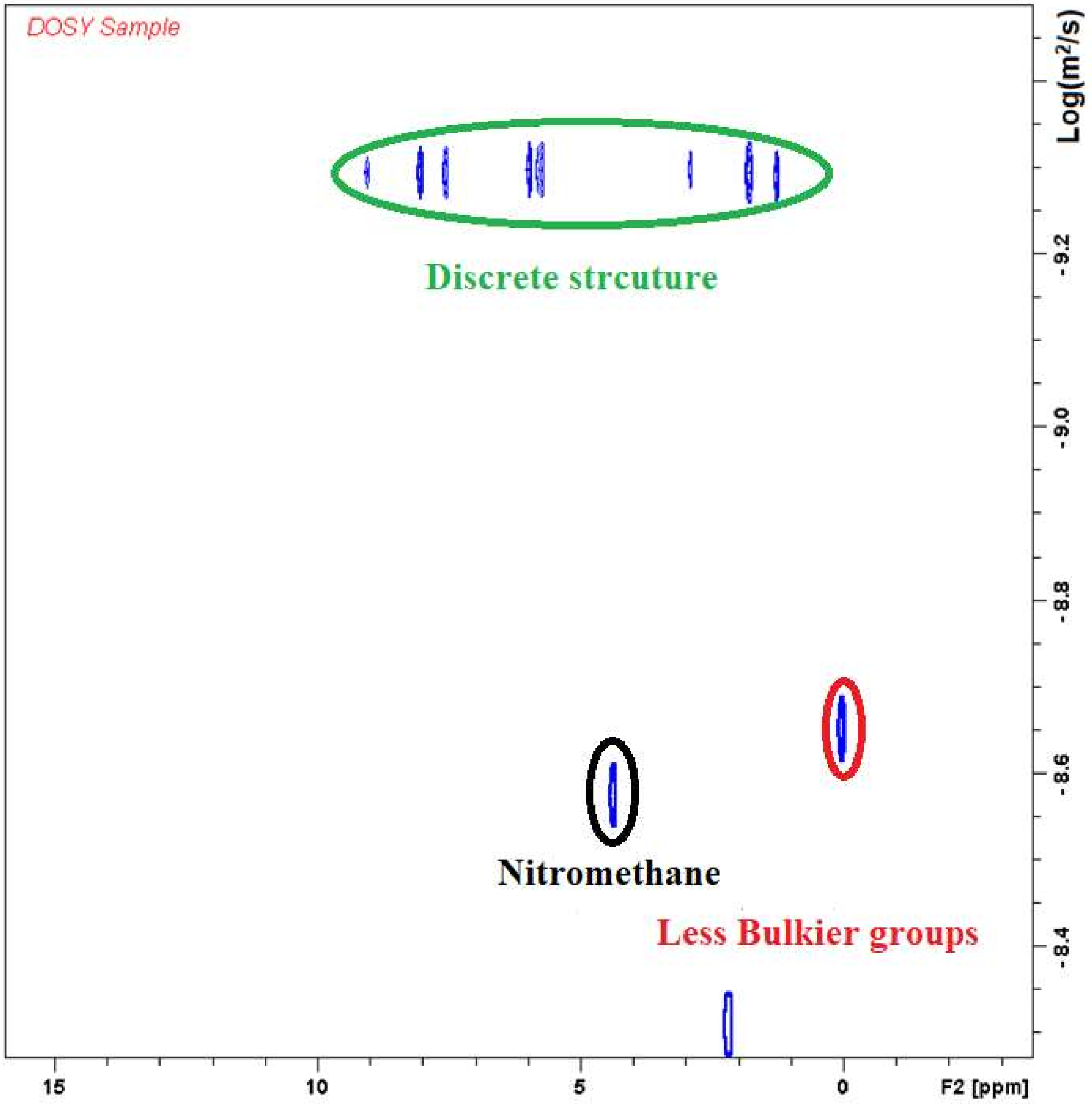

Figure S10. DOSY NMR spectrum of 2D Metalla-rectangle **3** in CD_3NO_2.

The DOSY experiment was conducted by using Innova spectrometer of a specific concentration in CD_3NO_2. The experiment clearly established the existence of only one species in solution with diffusion coefficient of 4.9×10^{-10} m^2s^{-1} at 25 ºC. As shown in Fig. S10, shows variable range of diffusion coefficient implies the presence of different moieties present in self-assembled structures.

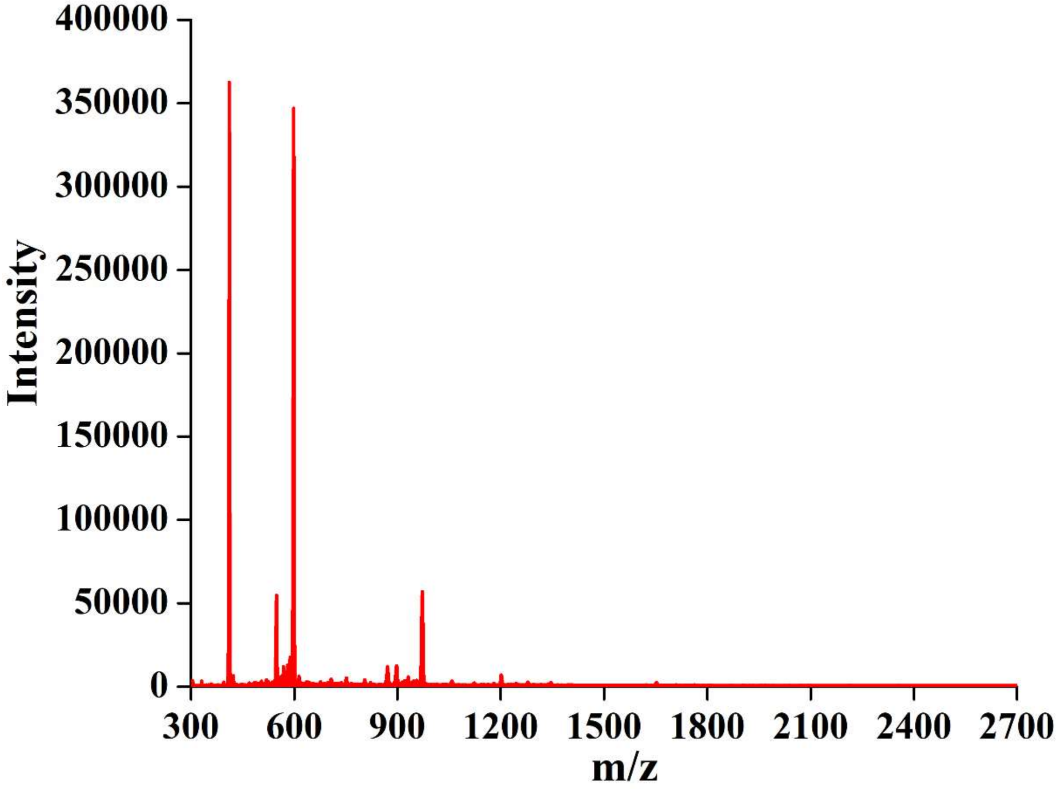

Figure S11. HR-ESI-MS Spectra of 2D metalla-rectangle **3** in methanol.

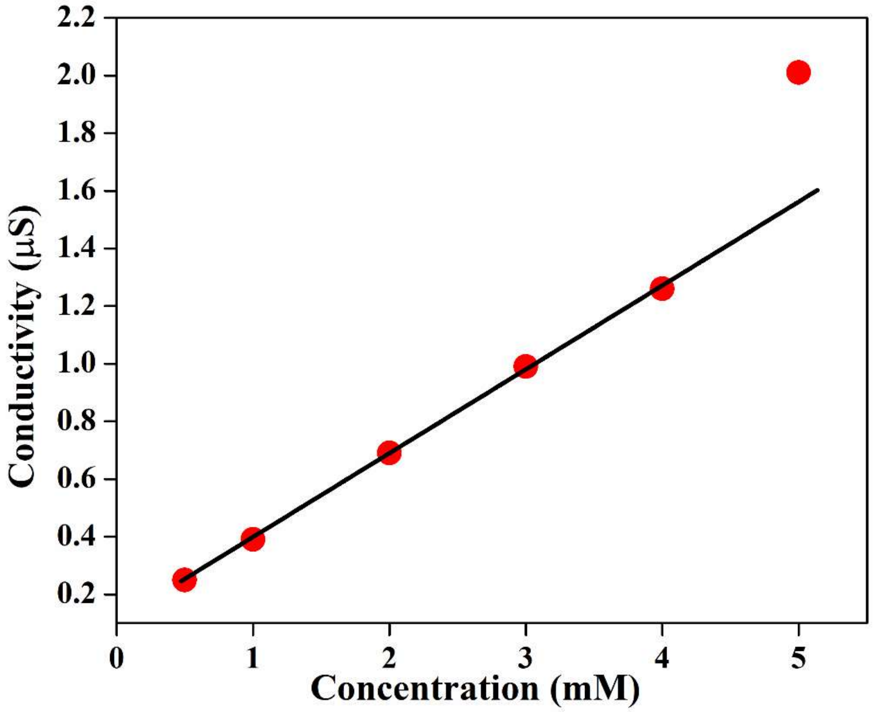

Figure S12. Variation in conductivity of 2D metalla-rectangle **3** with concentration.

Table S1. Elemental analysis comparison of ruthenium triflate complex and metalla-rectangle **3**.

Composition	C	H	N
$C_{28}H_{30}O_{10}S_2F_6Ru_2$	Found: 37.09<br>Experimental: 37.16	Found: 3.33<br>Experimental: 3.21	-------
$C_{78}H_{80}O_{22}N_8S_4F_{12}Ru_4$	Found: 40.69<br>Experimental: 41.79	Found: 3.19<br>Experimental: 3.60	Found: 5.12<br>Experimental: 5.00

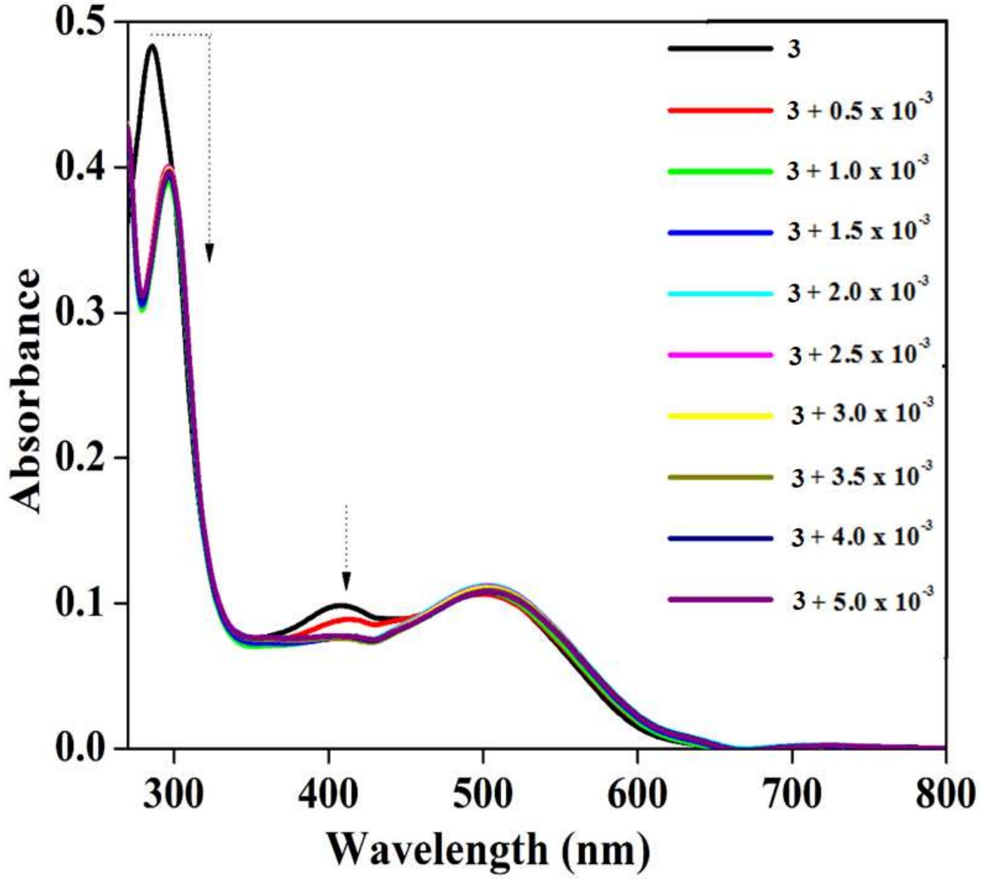

Figure S13. Change in absorbance of metalla-rectangle (**3**) upon addition of varying concentration of sodium oxalate.

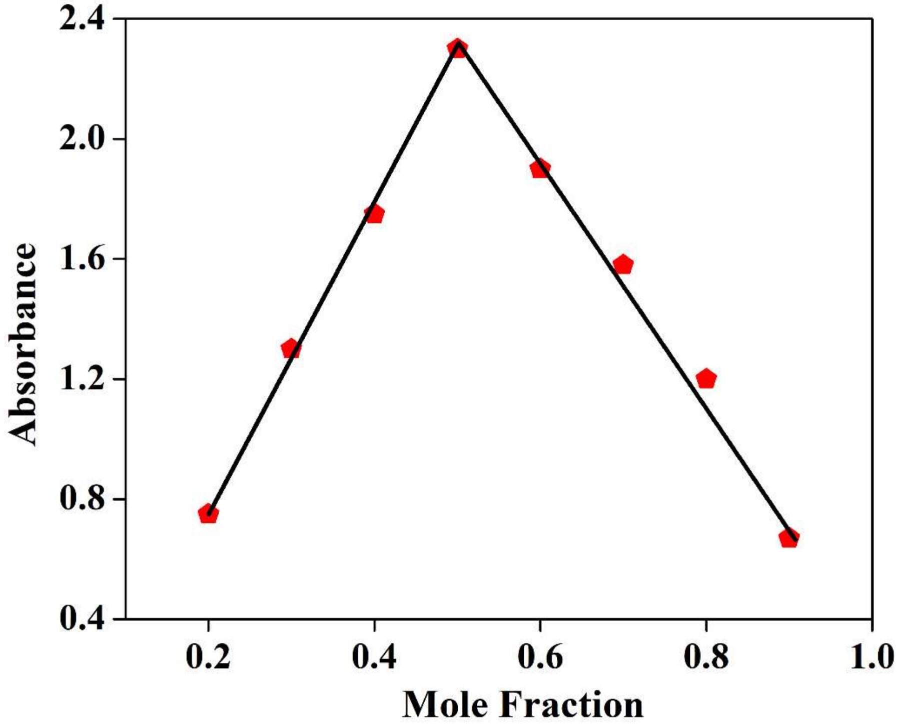

Figure S14 Job's plot of oxalate anion titrations with metalla-rectangle **3** showing 1:1 fitting curve.

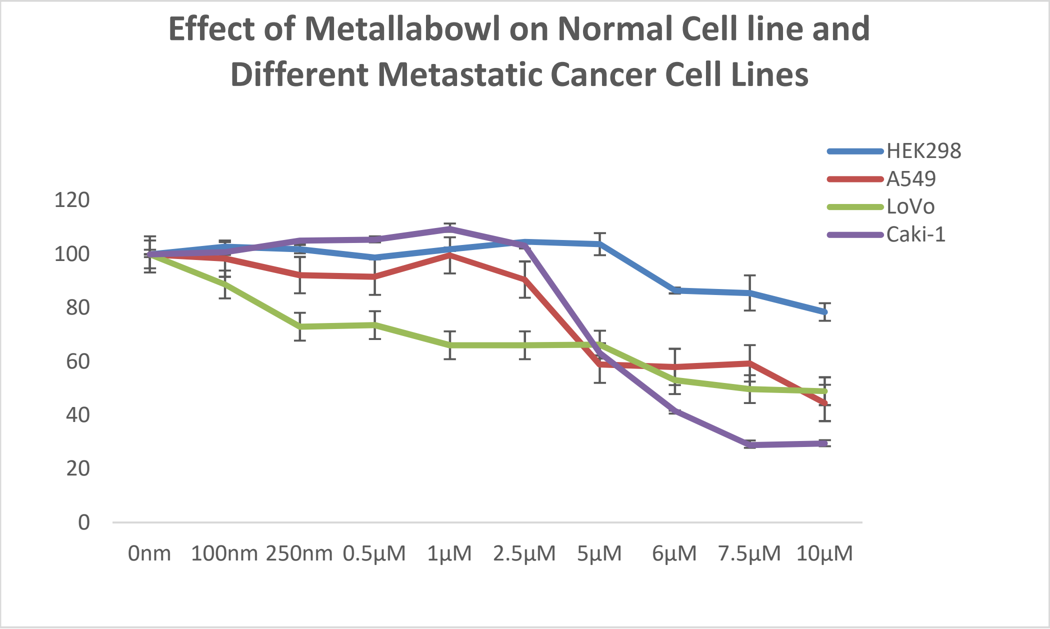

Figure S15: Effect of metalla-rectangle (**3**) on difference metastatic cancer lines and normal cell lines.
